# UNITE4TB: a new consortium for clinical drug and regimen development for TB

**DOI:** 10.5588/ijtld.21.0515

**Published:** 2021-11-01

**Authors:** M. J. Boeree, C. Lange, G. Thwaites, N. Paton, R. de Vrueh, D. Barros, M. Hoelscher

**Affiliations:** 1 Lung Diseases, Radboud University Medical Center, Nijmegen, The Netherlands; 2 Clinical Infectious Diseases, Research Center Borstel, Borstel, Germany; 3 Clinical Research Unit, Hospital for Tropical Diseases, Oxford University, Oxford, UK; 4 Global Health Portfolio, Utrecht, The Netherlands; 5 Lygature, Utrecht, The Netherlands; 6 Global Health, GSK, Brentford, UK; 7 Department of Infectious Diseases and Tropical Medicine, Munich, Germany

After decades of limited progress, the development of novel anti-TB medicines was revitalised at the start of the 21^st^ century through new initiatives and investments. This has resulted in innovations in treatment regimens thanks to new compounds becoming available and the re-purposing of existing drugs. However, progress has been relatively slow for a disease that, until the emergence of the COVID-19 pandemic, has remained the top infectious killer worldwide. Accelerated action is therefore necessary to reach the ambitious target of “ending TB” set by the WHO within its new Global Strategy (2016–2030).

We are now at the start of a new era. With financial support from the Innovative Medicine Initiative (IMI) in Europe and the German Federal Ministry of Research and Education (BmBF), the UNITE4TB Consortium will begin work in 2021. With a total budget of 185 million euros, 22 European academic institutions, 3 research institutions from the United States and South Africa, 2 European Federation of Pharmaceutical Industries and Association (EFPIA) members and 2 Associated Partners (APs) from Germany, this will be the largest collaboration on clinical TB drug development in the history of the European Union. Over the next 7 years, the Consortium will engage with up to 40 potential trial sites across the globe (including Europe, Asia, Africa and South America) to deliver innovative Phase 2 clinical trials that aim to speed the passage of new anti-TB drugs and regimens to definitive Phase 3 trials and regulatory approval.

The increasing clinical and public health challenge of multidrug-resistant TB (MDR-TB i.e., TB with resistance to at least the two best first-line drugs, rifampicin and isoniazid) has triggered renewed scientific activity in anti-TB drug development. In 2014, the European Medicines Agency (EMA) gave conditional marketing authorisations for pioneer compounds in two novel classes of anti-TB drugs, bedaquiline^1^ and delamanid.^2^ In 2020, the EMA^3^ and US Food and Drug Administration (FDA)^4^ approved the first-ever, all-oral BPaL regimen for extensively drug-resistant TB (XDR-TB) and treatment-intolerant and failing MDR-TB. However, a more integrated approach to anti-TB drug development is essential, facilitating collaborative critical paths for new combination regimens for TB, while simultaneously leveraging investments in both preclinical and clinical research capacity and de-risking Phase 3 clinical trials.^5^ Historically, to gain marketing approval for anti-TB drugs, large and expensive Phase 3 clinical trials have been required to demonstrate efficacy and safety of a new drug, or a new combination regimen, based on results from Phase 2 clinical trials. However, their success has been inconsistent, as exemplified by the fluoroquinolone trials (RemoxTB,^6^ RIFAQUIN^7^ and OFLOTUB^8^), and the partial disconnect between the results of the delamanid Phase 2 and Phase 3 trials.^9^ The success of the 4-month rifapentinemoxifloxacin regimen is a source of hope for TB regimen development,^10^ but it comes 21 years after the initial licensure of rifapentine by the US FDA.^11^ In 2018, a technical consultation organised by the WHO Global TB Programme acknowledged the limits of this traditional pathway and of the traditional endpoints used in Phase 2 trials to predict relapse-free cure with accuracy.^12^ It made a strong case for new approaches in TB treatment development using innovative Phase 2 and Phase 3 trial designs, relying on novel biomarkers and revised endpoints to assess in a shorter and more streamlined way new combinations of anti-TB drugs to be tested in pivotal trials.^13^

**Figure 1:**
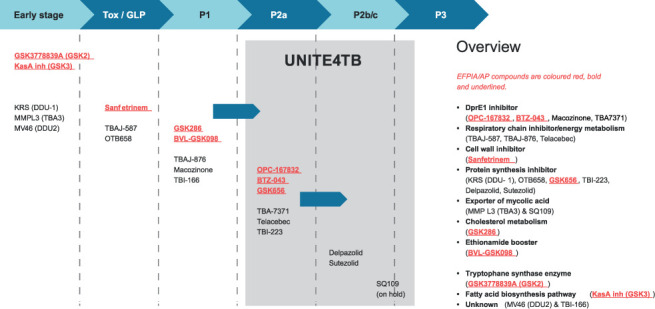
Global TB drug pipeline (October 2020), with UNITE4TB EFPIA/AP drug candidates in red in the online version/grey in print. EFPIA/AP = European Federation of Pharmaceutical Industries and Associations/Associated Partners.

The UNITE4TB Consortium will address the major challenge of defining new anti-TB drugs and regimens for the 21^st^ century. The consortium will create a platform for conducting innovative Phase 2A, and Phase 2B/C clinical trials that can reliably and efficiently identify, anti-TB drug regimens at the optimal durations with the best chance of success in definitive, large-scale, Phase 3 trials. The TB drug pipeline which will feed the Consortium’s clinical trials is presented in Figure 1. The complexity of developing novel regimens and reliable predictive biomarkers will be comprehensively and directly addressed to minimise operational barriers to co-development of compounds. Achieving this goal will facilitate fulfilment of one of the main unmet needs of the TB field: better-tolerated drug regimens of shorter duration that can treat TB with variable drug resistance patterns and avoid complications due to comorbidities. The critical challenge for global TB drug development over the next decade, which the UNITE4TB Consortium will address, is that the current approach to developing drug regimens likely to succeed in Phase 3 trials is failing. If we continue with current trial designs and endpoints, generally focused on the development of single drugs rather than regimens, it is estimated it will take 15–20 years to develop a new regimen of 3–4 drugs to treat TB.^9^ Given the scale of the TB public health problem, this is unacceptable. New approaches are required and our Consortium has the expertise, capacity and influence to change the paradigm of clinical TB drug development.

**Figure 2:**
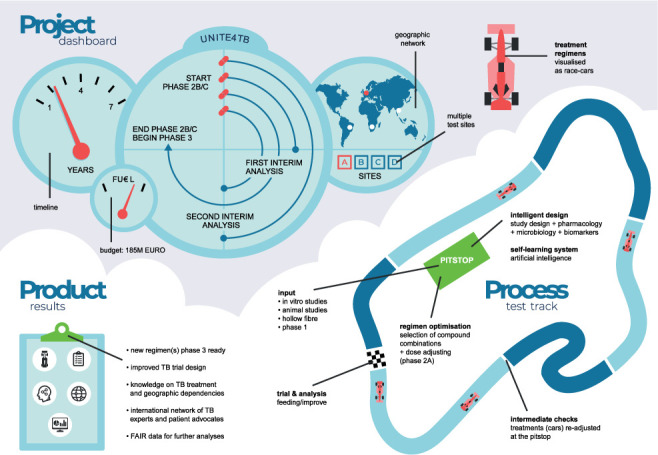
The UNITE4TB racetrack concept for the development of new drugs and regimens: the individual compounds are represented as essential car parts (e.g., tyres, engine, etc.). When ready, they enter the pitstop, where they are prepared to be incorporated into a car, or new treatment regimen. Potential regimens are launched onto the racetrack (the CT platform) to participate in the race (to select the best car or treatment regimen). FAIR = Findability, Accessibility, Interoperability, Reusability; CT =clinical trial.

The overarching concept for addressing the challenge is to develop drugs and regimens with a system analogous to a car racetrack (Figure 2). The individual drugs are depicted as essential *car parts*. When they are ready (i.e., meaning that they have established pre-clinical, Phase 1 and Phase 2A data), they enter the *pitstop*, where they are prepared to be incorporated into a *car*, or new treatment regimen. Data from Phase 2A trials will be particularly important, providing pharmacokinetics/pharmacodynamics (PK/PD) assessments that will indicate the likely role, effective dose and duration of administration for the individual drug in any given regimen. Then, potential regimens are launched into the racetrack (which is the clinical trial platform) to participate in the *race* to select the best *car* or treatment regimen. New *car parts* (drugs) and *cars* (regimens) may enter the race when ready. The treatment regimens can experience three fates: removal, improvement, or a win. Among the latter, the best, as assessed by their efficacy and safety, will be ready for a Phase 3 clinical trial.

To be successful, the UNITE4TB Consortium requires a wide and intense collaboration across many disciplines. The UNITE4TB Consortium has brought together global leaders in the areas of TB clinical trial design, execution of Phase 2A and 2B/C trials,^14^–^16^ PK/PD and pharmacometrics, microbiological surveillance, biomarker development and analysis, artificial intelligence and machine learning, digital adherence technologies, data management, privacy and sharing, dissemination and communication, ethics, community engagement, and biobanking. Within the next 7 years, the Consortium intends to have impact on a number of critical issues within TB drug development:

- Provide new tools and understanding on how to progress TB science for the discovery and development of new clinical candidates and combinations thereof across the research and development landscape, with special emphasis on innovative clinical trial design and the development of novel biomarkers;
- Develop imaging technology, biomarkers and pharmacogenomics, diagnostics and exploit artificial intelligence for the development of new clinical candidates and combinations;
- Enable the progression of potential new, safe, efficacious, shorter and affordable treatment solutions for TB patients worldwide, with the intent to improve the quality of life and life expectancy of patients;
- Contribute to the development of a vibrant global research environment, fostering private-public collaboration across EFPIA, academia, non-governmental organisations and small- and medium-sized enterprises and strengthening the competitiveness and industrial leadership of Europe;
- Provide a legal frame and agreement on intellectual property terms and exploitation, as a paradigm of public and private international collaboration in the development of combination regimes;
- Implement agreements with other consortia facilitating prompt data sharing and data exploitation to accelerate TB drug regimen development;
- Contribute to the EU’s ambition of being a ‘best practice region’ for addressing antimicrobial resistance, and profit from its medical capacity to individualise and implement into medical practice combination therapies addressing MDR- and XDR-TB.

Finally, the Consortium has the ambition to advance TB science and help the global community attain the 2030 United Nations Sustainable Development Goals and WHO 2035 End TB Targets, as well as the EU and European Member States’ commitments made through the UN high-level meetings on antimicrobial resistance and TB.

The spirit of cooperation and integration of a wide number of research partners and diverse research disciplines is a reflection of a global trend and is not limited to UNITE4TB. The TB field has been successful in developing novel public-private partnerships, with early dialogue with and between regulators, policy makers and donors, critical to keeping the promises made in the Millennium Development Goals. TB has been emphasised as a health priority by the G20 heads of states communique, and the first-ever UN High-Level Meeting on Ending TB. Several related activities were initiated in 2020/21, including the TB Drug accelerator and PAN-TB by the Gates Foundation (https://www.europeanpharmaceuticalreview.com/news/114186/global-collaboration-formed-to-develop-novel-treatment-regimens-for-tb/), IMI-ERA4TB (the pre-clinical sister of UNITE4TB; https://era4tb.org/) and IMI-PEARL (preparing the Phase 3 platform trial infrastructure in Europe; https://eu-pearl.eu/). The reapplication of the large US clinical trial networks (AIDS Clinical Trials Group network, https://actgnetwork.org/; and Tuberculosis Trials Consortium, https://www.cdc.gov/tb/topic/research/tbtc/) open a further opportunity to implement coordination and complementary co-operation in the field of TB drug development. The UNITE4TB research consortium will continuously update the TB community through publications in the *IJTLD* and other journals, describing its progress on this exciting journey.
